# Scalable, open-access and multidisciplinary data integration pipeline for climate-sensitive diseases

**DOI:** 10.12688/wellcomeopenres.24774.2

**Published:** 2025-11-15

**Authors:** Abhishek Dasgupta, Iago Perez-Fernandez, Tuyen Huynh, Cathal Mills, Rowan C. Nicholls, Prathyush Sambaturu, Marc Choisy, David Wallom, Tung Nguyen-Duy, Rhys P. D. Inward, John-Stuart Brittain, Sarah Sparrow, Moritz U.G. Kraemer

**Affiliations:** 1 Oxford Research Software Engineering Group, Doctoral Training Centre, University of Oxford, Oxford, UK; 2 Pandemic Sciences Institute, University of Oxford, Oxford, England, UK; 3 Oxford e-Research Centre, Department of Engineering Science, University of Oxford, Oxford, England, UK; 4 Oxford University Clinical Research Unit, Ho Chi Minh City, Vietnam; 5 Department of Statistics, University of Oxford, Oxford, England, UK; 6 Department of Biology, University of Oxford, Oxford, UK; 7 Nuffield Department of Medicine, University of Oxford, Oxford, England, UK

**Keywords:** data science, automated workflows, climate-sensitive infectious diseases, dengue

## Abstract

Climate-sensitive infectious diseases pose an important challenge for human, animal and environmental health and it has been estimated that over half of known human pathogenic diseases can be aggravated by climate change. While climatic and weather conditions are important drivers of transmission of vector-borne diseases, socio-economic, behavioural, and land-use factors as well as the interactions among them impact transmission dynamics. Analysis of drivers of climate-sensitive diseases require rapid integration of interdisciplinary data to be jointly analysed with epidemiological (including genomic and clinical) data. Current tools for the integration of multiple data sources are often limited to one data type or rely on proprietary data and software. To address this gap, we develop a scalable and open-access pipeline for the integration of multiple spatio-temporal datasets that requires only the declaration of the country and temporal range and resolution of the study. The tool is locally deployable and can easily be integrated into existing climate-disease-modelling applications. We demonstrate the utility of the tool for dengue modelling in Vietnam where epidemiological data are legally required to remain local. We include a pipeline for bias correction of climate data to enhance their quality for downstream modelling tasks. The Dengue Advanced Readiness Tools-Pipeline empowers users by simplifying complex download, correction, and aggregation steps, fostering data-driven discovery of relationships between infectious diseases and their drivers in space and time, and enhancing reproducibility in research. Additional modules and datasets can be added to the existing ones to make the pipeline extendable to use cases other than the ones presented here.

## Plain language summary

Most human infectious diseases are affected by climate. Therefore, understanding how climate and other rapidly changing factors like land use, population behaviour, and socio-economic conditions influence the spread of these diseases requires combining many different types of data. However, tools that enable researchers and policy makers to bring together and use this data remain limited, difficult to use, or rely on software that is not freely available.

To address this gap, we developed a new, open-access software pipeline that helps users collect, clean, and prepare different types of data—such as weather, socio-economic, and health data—for analysis. The pipeline works with data across different locations and time periods, and only requires users to specify the country and timeframe they are interested in. It is designed to be easy to use and can be run on local computers, which is especially important in places where health data is not allowed to leave the country. We deployed the pipeline using data on dengue fever in Vietnam. By making the process simpler and more transparent, we hope to support faster and more reliable responses to climate-related health threats.

## Introduction

Climate-sensitive infectious diseases represent a growing public health challenge with changes in weather and climate that can create conditions that influence disease transmission ^1– 4^. Recent estimates suggest that over half of known human pathogenic diseases may be aggravated by climate change, underscoring the urgent need for enhanced understanding of the mechanisms linking environmental change and disease dynamics ^4^. While climatic and weather variables such as temperature, precipitation, and humidity are well-recognised drivers of vector-borne diseases and respiratory viruses ^5^, non-climatic factors including demographic, socio-economic conditions, human behavior, and land-use changes also play critical roles ^6– 9^. Understanding how these diverse drivers interact requires an integrative approach that bridges multiple scientific disciplines and data types ranging from tabular to satellite imagery.

Despite growing recognition of the need for interdisciplinary approaches, existing tools for integrating spatio-temporal data relevant to climate-sensitive diseases remain limited ^9, 10^. Many platforms focus on specific data types, such as Google Earth Engine for satellite imagery ( https://earthengine.google.com/), or rely on proprietary datasets and software, such as Atlas AI ( https://www.atlasai.co/). This fragmentation prohibits comprehensive analyses by requiring researchers to manage disparate platforms and potentially apply complex and time-consuming preprocessing steps manually ^10^. These challenges are further compounded when working with epidemiological data that may be sensitive or legally constrained from leaving local contexts requiring flexibility for tools to be easily locally deployable.

To address these limitations, we developed the DART (Dengue Advanced Readiness Tools, https://www.dartdengue.org/) pipeline, an open-access, locally deployable system designed to streamline the integration of diverse spatio-temporal epidemiological, socio-economic, and climatic datasets. After defining the spatial extent of a study area (country ISO3 code) with the desired administrative unit and the time frame of the study, researchers can automatically access, download, and preprocess environmental, and socio-economic data for joint downstream analyses at daily, weekly or monthly timesteps. The pipeline supports data integration of epidemiological data, enabling incorporation into climate-disease modelling applications and more broadly real-time modelling tasks.

We demonstrate the capabilities of the DART pipeline through a case study on dengue transmission modelling in Vietnam, where epidemiological data must remain locally stored. Comprehensive documentation and user guides are available at: https://dart-pipeline.readthedocs.io/en/latest/.

## Methods

**Figure 1.**
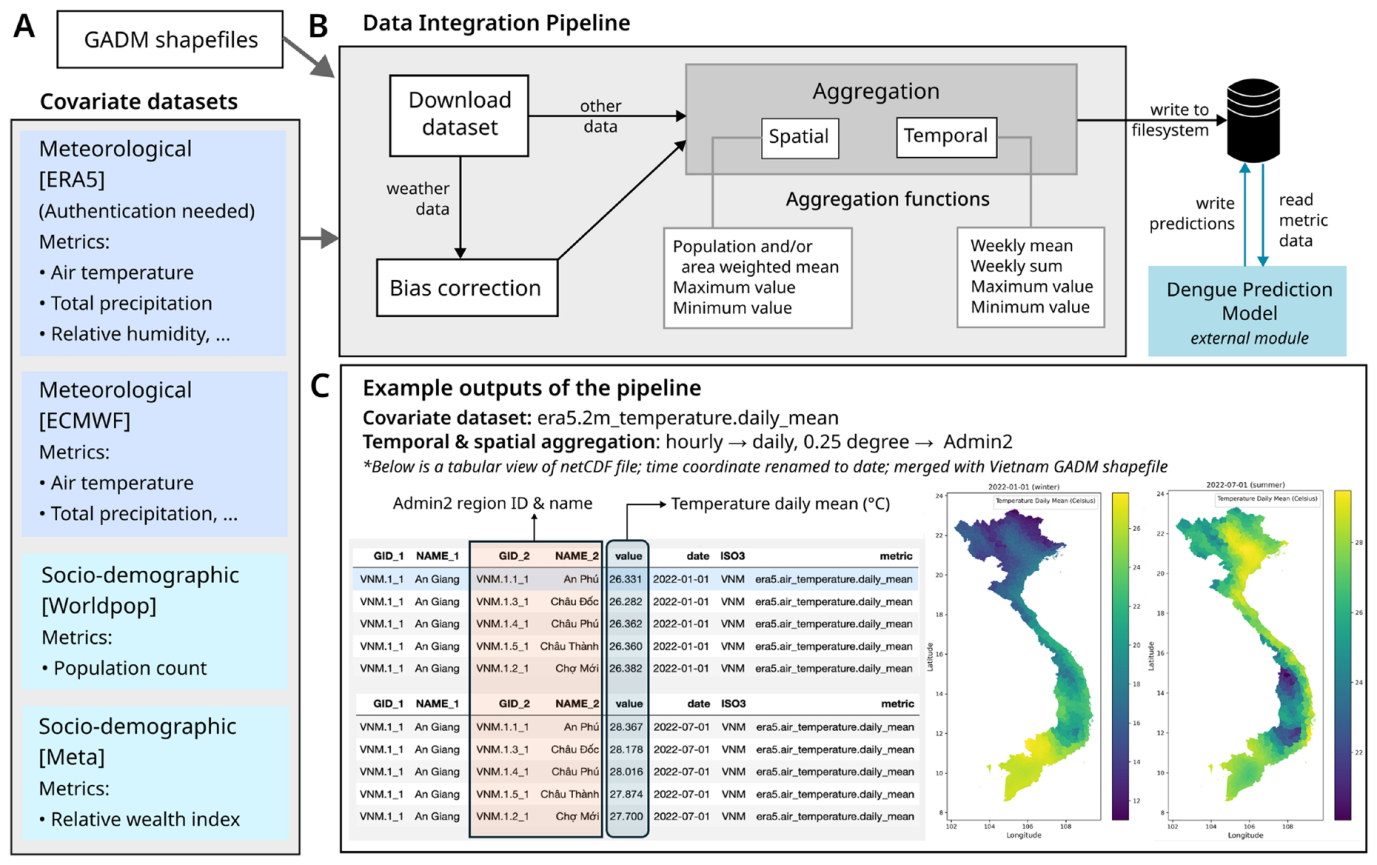
Workflow of the Data Integration Pipeline and Example Outputs. **A**) Overview of the input datasets used in the integration pipeline. These datasets include various covariates associated with dengue incidence, categorised into meteorological and socio-demographic factors. For each dataset, a selection of relevant variables, data sources, and access restrictions, where applicable, is provided. Additionally, shapefiles from the Database of Global Administrative Areas (GADM) were used to define the geographical boundaries of administrative units at multiple levels (e.g., admin2 and admin3) considered in the pipeline. **B**) The integration pipeline consists of multiple sequential steps. First, datasets containing the relevant variables are downloaded from their respective sources. A dedicated sub-pipeline corrects weather data bias using quantile mapping technique. Finally, spatial and temporal aggregations are performed at the users predefined administrative levels. Spatial aggregation methods—such as population-weighted or area-weighted means, maximum, and minimum values—are applied based on the nature of each variable, using GADM shapefiles for administrative levels admin2 and weighted using Worldpop population data. Similarly, temporal aggregation methods, including weekly mean, sum, maximum, and minimum values, are computed. The processed data is then stored in a structured schema in the standard netCDF file format, which includes region ID, datetime (corresponding to the variable’s weekly value), variable metadata, and the aggregated value. Further, the aggregated dataset is incorporated into dengue modelling for further analysis, and the model’s outputs are subsequently stored in the database. **C**) This panel illustrates example outputs using the air temperature at 2m variable from the ERA5 dataset. The data is temporally aggregated (see Table S1 for methods) and then spatially aggregated to the admin2 level. The tables display the daily mean air temperature for admin2 regions on two specific dates: January 1, 2022, and July 7, 2022. Corresponding maps on the right visualise this data, where lighter shades represent higher daily mean temperatures, and darker shades indicate lower temperatures, providing the spatial distribution of temperature variations.

The variables included in our pipeline were selected primarily for forecasting dengue cases ^11, 12^, based on their previously estimated impacts on disease transmission, influencing and modulating vector and pathogen survival, development, and reproduction, and human susceptibility and exposure ^13– 17^. These variables have also been used in previous forecasting studies for climate-sensitive diseases other than dengue (e.g. West Nile Virus ^18^). Table 1 shows the current challenges for robust analyses of climate-sensitive diseases and the functionality of the proposed digital tool. Figure 1 shows the workflow involved in the pipeline from initial download to final storage of files in netCDF format.

**Table 1.** Challenges in the generation and execution of multidisciplinary data integration pipelines in order of consideration.

Process	Challenges	*DART* pipeline functionality
Dataset identification	• Availability of a large number of covariates, including multiple for the same variables • Requires domain expertise from multiple different domains (epidemiological, climatic, environmental, socio-economic)	• Curated set of covariates that work well together • Domain expertise is not needed as all variables are aggregated to a unified schema for easy comparison
Data download	• No automated download functionality across different datasets and domains • Issues with data licensing and attribution	• Single interface to download data across different domains and process to an unified schema • With a few exceptions, curation from public data sources and documented attribution
Data aggregation	• Raw data are rarely at the temporal and spatial scales needed for downstream applications • Aggregation conventions differ across domains requiring human input, capabilities and judgement • Bespoke code needed for aggregating spatio-temporal data	• Pre-processing steps in the pipeline ensure appropriate temporal and spatial resolutions, with aggregation to daily and weekly levels in temporal scale • Aggregation of data to administrative units using appropriate aggregation methodology for each variable. Pre-selected aggregation methods that can be extended or replaced • In addition to standard aggregation functions (sum, mean), population weighting of meteorological variables is performed as it is often more relevant for infectious disease research
Data validation	• Large data pipelines are often error-prone which could impact downstream applications	• Unit and integration testing, along with visual checks by experts
Data integration	• Integrating data across epidemiological, climate, environmental, and socio-economic domains is challenging due to differences in spatial and temporal definitions, references, coverage and resolutions • Epidemiological data are often sensitive and cannot be shared outside of specific agencies	• Unified data storage in netCDF with (time, region) coordinates and annotated with CF-compliant and DART specific metadata that allows comparison to private datasets that cannot be included within the public pipeline

**Observational data:** In this study we used reanalysis data to represent the weather conditions observed in a specific period of time. Reanalysis data shows an accurate estimate of the state of the atmosphere/ocean for a particular time step, and this dataset is obtained by combining observations and short range weather forecasts (1–3 days in advance) computed by numerical weather prediction models ^19, 20^. Even though reanalysis data shows a very accurate representation of the weather conditions, they might deviate from the observations for some regions ^19^, affecting the forecasting skill of the Dengue prediction model. Hence applying a bias correction technique will reduce the deviation between observations and predictions. Bias correction techniques are applied to correct precipitation data using a method called quantile mapping ^21, 22^. This technique is based on the interpolation of the quantile points from a target dataset to a reference dataset so the corrected distribution has statistical properties closer to the observations. Here, we used Vietnam gridded precipitation data ^23^ as the reference and ERA5 ^20^ and applied the correction following a daily timestep. We also replace outliers above 99th percentile in the reference data by the corresponding 99th percentile value as quantile mapping struggles at correcting extremely high values of daily precipitation, inflating extreme precipitation values obtained in the correction ^21^. This way we avoid the manifestation of anomalously high precipitation values in the correction.

**Socio-economic data.** In addition to observational data, we integrated Meta’s relative wealth index (RWI) data into the pipeline following the processing steps outlined in ( https://dataforgood.facebook.com/dfg/docs/tutorial-calculating-population-weighted-relative-wealth-index). The RWI data are provided by Meta as tabular point-level estimates indexed by quadkeys, each representing a ~2.4 km grid cell. Each record includes latitude and longitude coordinates corresponding to the grid cell centroid, enabling spatial mapping and integration with other geospatial datasets. In our implementation, RWI values are aggregated to administrative units with population-weights (mean), with Meta’s population density data serving as weights. Latitude and longitude are used primarily for spatial referencing and visualization, while the quadkey identifier ensures consistent alignment between RWI and population layers during aggregation. The framework also supports replacing Meta’s population data with census-derived population grids to calculate population-weighted RWI or incorporate alternative socioeconomic indicators.

**Weather forecasting:** In addition to using reanalysis data, we also included future weather forecast data up to two weeks ahead. Weather forecast data is obtained from the European Centre for Medium-Range Weather Forecasts (ECMWF), specifically from the ensemble prediction system (hereafter ECMWF weather forecast model). ECMWF weather forecast model is a probabilistic prediction model that estimates the most probable state of the atmosphere in the future while quantifying the uncertainty of the predictions up to 15 days in advance. Nonetheless, the further we go into the future, the more uncertain the forecast becomes, hence weather forecast data is often post-processed to correct possible biases so it can be more useful for applications. Prior studies have shown that the weather variables that are most linked to Dengue incidence are temperature, relative humidity and accumulated precipitation, and early results from DART showed that applying quantile mapping to raw weather forecast data increases the reliability and accuracy of the predictions beyond 10 days ^22, 24^. We also applied quantile mapping to calibrate ECMWF weather forecast data, using ERA5 data as reference. The correction is applied separately to the weather forecast 11 ensemble members (10 perturbed + 1 control).

**Figure 2.**
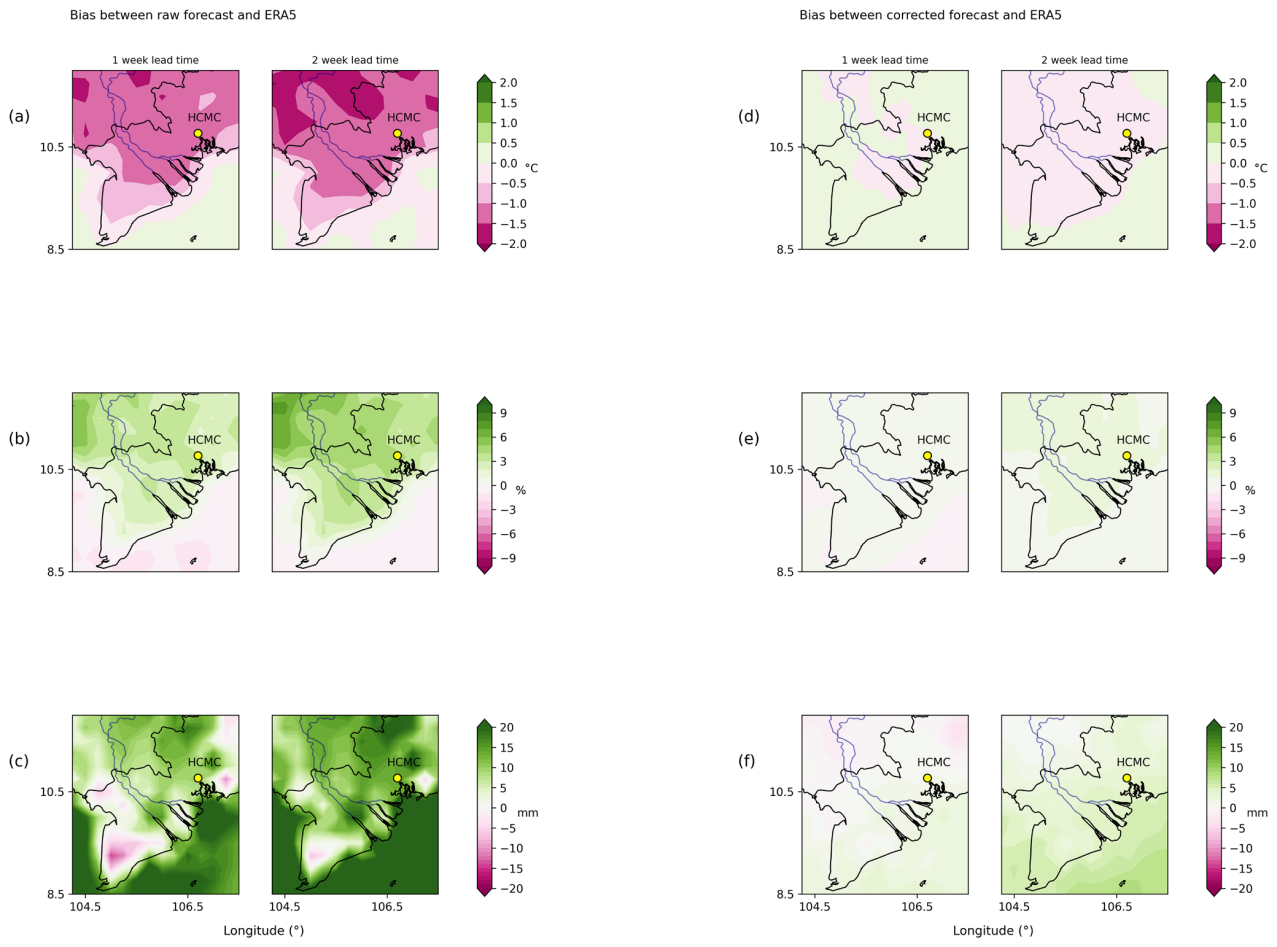
Bias between raw and corrected ECMWF weather forecast model against ERA5 in South Vietnam for 2 metre temperature (Figure **2a**, **d**), relative humidity (Figure **2b**, **e**) and total precipitation (Figure **2c**, **f**) between 1–2 weeks in advance between 2004–2020. Figure **2a** ( **2d**) shows results for raw (corrected) 2 metre temperature, **2b** ( **2e**) for raw (corrected) relative humidity and **2c** ( **2f**) for raw (corrected) accumulated precipitation.

In the following couple of figures, we show the effect of bias correction on weather variables in a region around Ho Chi Minh City, Vietnam (HCMC), which is our area of interest for the pipeline implementation. Figure 2 shows the mean difference (or bias) between raw ( Figure 2a–c) and corrected ECMWF weather forecast model estimates ( Figure 2d–f) against ERA5 for 2 metre temperature, relative humidity and total precipitation between 1–2 weeks in advance. Raw ECMWF forecast model predictions show a systematic cold bias, underestimating 2 metre temperature by ~2 degrees ( Figure 2a). Mean relative humidity does not appear to vary much from observations (it just shows a small positive bias) ( Figure 2b). In the case of accumulated precipitation ( Figure 2c), there is a positive bias (the model overestimates precipitation) around 20 mm in the coastal/oceanic regions and slightly less in HCMC city. After applying the quantile mapping technique, the biases between observations and corrected forecasts ( Figure 2d–f), are practically negligible.

**Figure 3.**
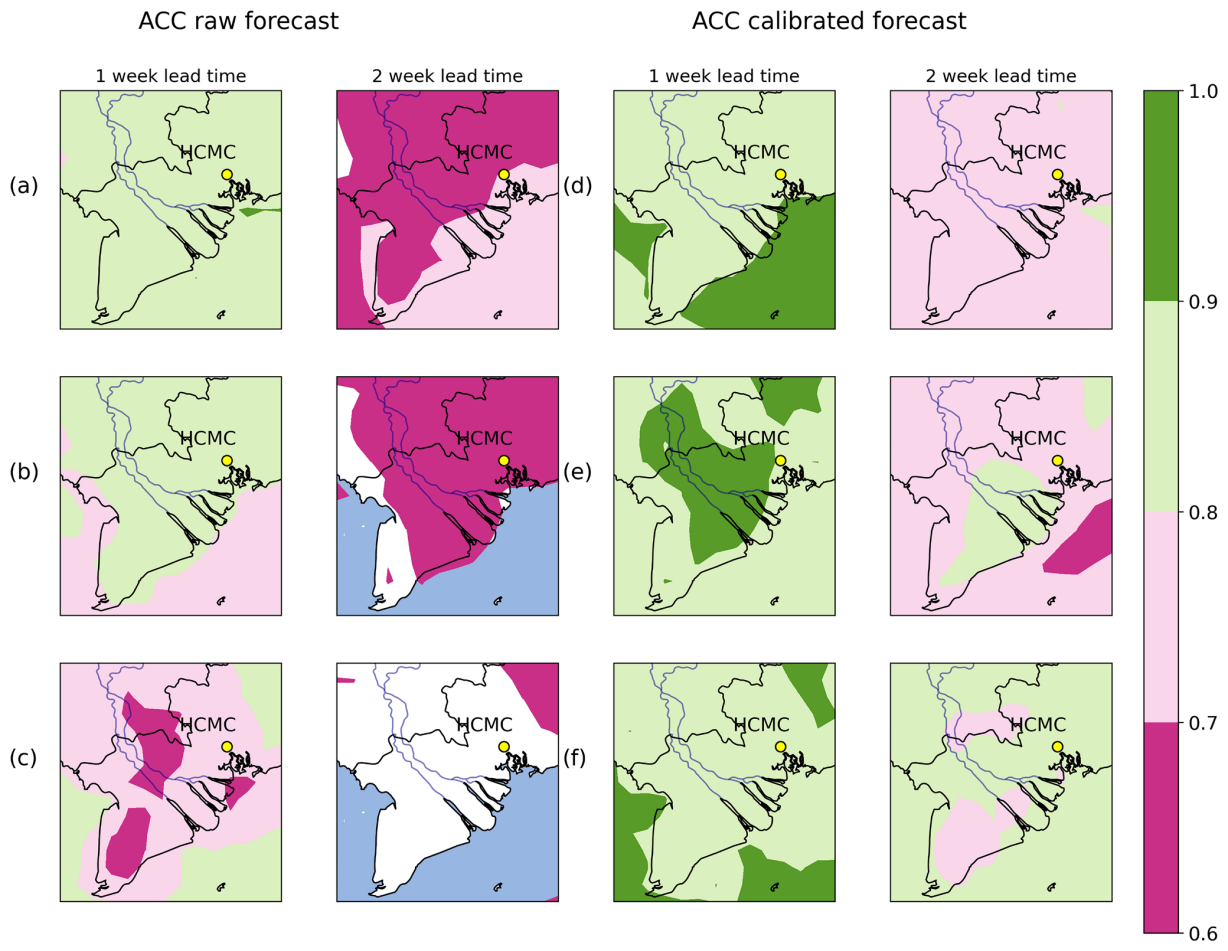
Anomaly correlation coefficient (ACC) for the region of South Vietnam for lead weeks 1–2 for uncalibrated ( **a**– **c**) and calibrated ( **d**– **f**) ECMWF weather forecast model and ERA5 data between 2008–2024. Figure **3a** ( **3d**) shows results for raw (corrected) 2 metre temperature, **3b** ( **3e**) for raw (corrected) relative humidity and **3c** ( **3f**) for raw (corrected) accumulated precipitation.

Another example of the performance of the calibration can be seen in Figure 3, where we show the anomaly correlation coefficient (ACC), between raw ( Figure 3a–c) and corrected forecast data ( Figure 3d,f). ACC quantifies the ability of the forecast model to predict values that deviate from the climatological mean. These climate anomalies should be accurately modelled as it is especially important for climate-sensitive diseases such as dengue. A recent study ^25^ suggested extreme weather events can lead to increased dengue risk, and another ^26^ identified how climate anomalies can be used to predict long-term trends of dengue. When ACC >0.6 the weather forecast is considered skillful. Here, we used ERA5 as reference data; raw ECMWF data for the first lead week of prediction shows ACC values around 0.8–0.9 for 2 metre temperature for the first week, and manages to remain skillful to the 2nd week near HCMC ( Figure 3a). In the case of relative humidity ( Figure 3b), results are similar to 2 metre temperature, with the difference that by the 2nd week the forecast is barely skillful. By contrast, when we measure the ACC using total precipitation, we find the lowest values during the 1st week of forecast (between 0.6–0.7 and in some areas we have no skill), and there is practically no skill at the 2nd forecast week ( Figure 3c). After applying the quantile mapping technique ( Figure 3d–f), we generally obtain higher values of ACC for the first week for every variable, (ACC values between 0.8–1.0). Forecast skill is improved 2 weeks ahead for all variables in South Vietnam, although for relative humidity ( Figure 3e) the skill is slightly lower compared to the other variables, but still well above the threshold (ACC>0.6).

Results from Figure 2 and Figure 3 show that the quantile mapping technique extends the reliability and accuracy of the forecast up to 2 weeks in advance, hence we applied this technique to correct 2 metre temperature, relative humidity and total precipitation for the weather forecast in order to obtain more accurate predictions of these variables.

ECMWF only makes ERA5 data available with a lag of 5 days which results in a 5-day gap between observations and the current date. In order to fill this 5 day gap critical for decision making, we obtain the weather forecast that initialises 7 days before the current date (t) and retain data that goes up to day t+7. We then perform bias correction on this weather forecast, aggregate daily forecasts into a one week prediction, and use this as a “pseudo-observation”, inputting it to the dengue prediction model for the following week.

**Spatial aggregation:** The climatic weather variables are stored in a spatio-temporal raster, while administrative unit boundaries are polygons (see Figure S1). We performed temporal and spatial aggregation to the administrative level that matches the resolution of the epidemiological data. Due to heterogeneity in population distributions within administrative units, we performed population weighting at 1km x 1km resolution. This aggregation process was performed using WorldPop population count rasters, ERA5 weather rasters and administrative boundaries from GADM ^27^. Users can optionally provide their own shapefiles via a custom configuration. The process follows the following six steps:

(i) All rasters are projected onto EPSG:4326 (standard latitude/longitude, WGS84 datum)

(ii) All raster files are cropped to the extent of the administrative units of interest (regions can be specified by the user)

(iii) Rasters are resampled to match the resolution of the population raster (in our case 1km x 1km but can be user defined with a custom function)

(iv) Each raster cell’s weighting is calculated according to a formula that includes the coverage fraction of each raster cell with the administrative boundary polygon from the shapefile, with weights proportional to the spherical area in square kilometres.

(v) Each raster cell is weighted by the population cell value, normalised by each polygons total population

(vi) Cell values are aggregated to the administrative units using either the population weighted mean or area weighted sum for instantaneous and accumulative variables respectively.

**Temporal aggregation:** Covariates have varying spatial resolutions with climate variables downloaded at hourly timesteps and aggregated to daily or weekly mean, maximum or minimum values per pixel. After the temporal aggregation, we perform spatial aggregation to a user-defined administrative level. We use the ISO-8601 standard for date ( https://www.iso.org/iso-8601-date-and-time-format.html) with weeks starting on Mondays.

**Data dictionary:** All output data are stored as netCDF files with one netCDF file per data source (historical ERA5 weather data, ECMWF forecast data and socio-demographic data). Metadata are stored as netCDF attributes and adhere closely to CF conventions ( https://cfconventions.org). Each netCDF has variables that are indicated by CF-compliant or commonly used short names (such as t2m for air temperature at 2m, and r for relative humidity). The following metadata attributes are present. Each variable in a netCDF file is defined by (time, region) coordinates.

**Global attributes:**

- DART_region: Global netCDF attribute, describes the region for which the zonal statistics was performed. This includes the region or ISO3 code, the shapefile column used to derive coordinates for ‘region’ (in our case, GID_2, the unique ID for GADM administrative level 2; user specified ID in case of custom configuration), the timezone (used for timeshifting hourly ERA5 data) and geospatial extents as a bounding box.

**Coordinates**:

- time: time coordinate for aggregated variable value (mean for instantaneous, sum for accumulative variables) until the next time step
- region: unique ID that denotes the administrative region over which zonal aggregation was performed

**Variable attributes:** These attributes are CF-compliant

- long_name: Description of the variable
- units: Name of the unit, as recommended by CF conventions *udunits* package ^28^
- cell_methods: indicating the temporal aggregation steps
- valid_min (optional): minimum valid value
- valid_max (optional): maximum valid value
- standard_name (optional): CF-compliant standard name if one exists

**Software Architecture:** The pipeline is written in *Python 3* and depends on well-known libraries in the geospatial Python ecosystem such as *rasterio* and *xarray* for raster manipulation, and *geopandas* and *shapely* for shapefile processing. To ensure reproducibility, we use a lock file which pins package versions, preventing package upgrades from breaking the pipeline; we will also attempt to keep the pipeline updated with the latest major versions of underlying dependencies. We chose *Python* for its extensive library support for data science and machine learning. We also provide a command line interface to make our pipeline interoperable with other tools and programming languages. We provide a user-friendly method to download data, with user prompts in cases where authenticated access is required, such as for ERA5 open data from ECMWF. Data are downloaded into standardised locations described in the documentation. Data processing is performed as described in the Methods section, where we use a helper library which we authored and called *geoglue* (available at https://github.com/kraemer-lab/geoglue), that enables higher level geospatial operations in *Python* compared to those offered by *rasterio*. For climate data, resampling to the target resolution of the population raster is performed by the Climate Data Operators library using the *remapbil* operator for bilinear interpolation (for instantaneous variables) and the *remapdis* operator for distance-weighted average remapping (for accumulative variables). Zonal statistics is performed using the *exactextract* package using operations suitable for each variable (weighted_mean for instantaneous, area_weighted_sum for accumulative variables, Table S2). After performing zonal aggregation, we annotate the data with appropriate metadata as described above.

Compared to prior packages for zonal aggregation in *Python*, such as *rasterstats*, the dependency in *geoglue* that we use for zonal aggregation, *exactextract*, can use the overlap fraction of a raster cell with a polygon, rather than entirely including or omitting a raster cell based on partial overlap which gives better results. We compute the weighted mean or area-weighted sum for each administrative region. As an illustration for this manuscript, we demonstrate the pipeline functionality for Vietnam, aggregating to Global Administrative Level 2 (i.e. district-level subdivisions, henceforth called “district”, n = 710 ^27^.

**Data architecture:** We store output data in flat files in the open-source and well-documented binary netCDF file format. We chose netCDF compared to the ASCII text file formats such as CSV due to its widespread use in climate data processing and for its rich metadata support. Libraries such as *xarray* and extensions of netCDF such as *zarr* and *icechunk* also enable future scaling to larger datasets. Storing data in a well-documented binary format along with libraries like *xarray* makes it possible to only work with a portion of the data and allow easy reshaping and resampling of the data. Using flat files enables scaling the solution from a users' laptop to the data center as data size and computational complexity of running the pipeline increases. It also enables simpler parallelization for large source datasets; by writing to a shared filesystem (or network data store), scripts can run in parallel on a laptop or use HPC infrastructure to parallelise fetching and processing data.

**Testing and validation:** We perform extensive unit testing of our code. Some of the data transformations rely on well-tested tools written in C++ such as Max Planck's Climate Data Operators and GDAL (depended upon by *rasterio*). For such cases, we rely on snapshot and regression testing to ensure the results are reproducible. Unit tests are run automatically on every code change using continuous integration with GitHub Actions. Regression testing is performed using cached data. Data transformations for a subset of climate variables (temperature and precipitation), as well as all socio-demographic variables are manually validated by experts in climate data. Range validation is performed (Table S3) and valid_min and valid_max attributes set on netCDF variables to indicate permissible ranges to downstream applications.

**Extensibility:** DART-Pipeline is written to be extensible and adaptable for future work that includes additional variables. Our code is open-source under the MIT license ( https://opensource.org/license/mit), allowing others to build upon our work. The bias-correction component is available as a separate module ( https://github.com/DART-Vietnam/dart-bias-correct) as it is GPL-3.0 licensed ( https://opensource.org/license/gpl-3-0) due to a dependency. The forecast correction component is available as part of another repository ( https://github.com/DART-Vietnam/dart-runner) that depends upon dart-bias-correct and the main DART-Pipeline. In addition, we have refactored out foundational components that are of use beyond the pipeline, such as manipulating rasters in memory, performing zonal statistics with a shapefile, and fetching reanalysis data from ECMWF into its own open-source package *geoglue* ( https://geoglue.readthedocs.io) that users can use for tasks beyond this pipeline. Within the pipeline, code for each data source is in a single Python submodule, allowing extensibility to other climate, socio-demographic and epidemiological datasets in the future.

**Accessibility of data sources:** All primary data sources for DART Pipeline are open access, either through direct downloads or via a free-to-use API. Data sources required for bias correction are not open access – we provide links to instructions to get access. Users can run the pipeline without bias correction with entirely open-access data. For the purposes of reproducibility, we maintain a private AWS S3 bucket with source and intermediate files created during the processing of the pipeline for the real-time data. While these data cannot be shared, users can reproduce the output by re-downloading data and running the pipeline. ERA5 reanalysis data can be obtained by registering and logging in. To enable easier access, we prompt the user to create API tokens and allow easy entry of these tokens when the pipeline is run, along with documentation about licensing and redistribution.

**Installation and compatibility:** DART-Pipeline has been tested on macOS Apple Silicon, macOS x86_64 architecture and Linux x86_64. The minimum Python version supported is 3.11. Users also need to download the Climate Data Operators program ( *cdo*) separately which is required by the resampling step ^29^. A step-by-step guide on how to install and use the pipeline is provided in our documentation.

**Use case**: For the purpose of testing the pipeline, we selected a comprehensive list of meteorological variables from ERA5 and socio-demographic data from WorldPop and Meta and processed them for a dengue forecasting pipeline in Vietnam. Dengue epidemiology in Vietnam is highly heterogeneous with hyper-endemicity and year-round transmission in the southern part of the country and long-term emerging and high seasonality in the northern part ^30^.

We chose HCMC as the exemplar study region. HCMC is located in the southern part of the country. It is the economic capital and most populous city of Vietnam, with large seasonal dengue outbreaks during the rainy season. Like many large urban centres in endemic areas, outbreaks vary in size and peak timings from year to year depending on climate and socio-demographic factors ^31^. Dengue control in the city relies largely on targeted vector control managed through the HCMC Center for Disease Control (HCDC); however, due to resource constraints, control efforts are carried out in response to local outbreaks in the city and not proactively. In such settings, forecasting at the spatial level for which vector control and hospital patients are managed, i.e. district level, can potentially improve the effectiveness of these vector control measures.

As the epidemiological data cannot be shared outside of Vietnam due to legal data sharing restrictions, we developed the pipeline to be locally deployable and open-source to facilitate adoption in the local context. The epidemiological data for HCMC are available from 2000 to 2022. The data, which contains residential addresses of admitted patients, is then aggregated to the district level. The meteorological and socio-economic data listed in Table S1 were aggregated to the same resolution using the tool described in this paper.

Data from 2020 to 2022 are excluded for subsequent analyses because (i) COVID-19 lockdowns in the city during 2020 and 2022 affected dengue virus transmission and reporting due to healthcare resources that were diverted elsewhere (Figure S2), and (ii) a new sub-city (Thu Duc city) was established at the end of 2020 ^32^, rearranging the spatial boundaries of districts within HCMC. Data from 2000 are also excluded due to concerns of data reporting quality and to keep temporal ranges in-line with other data. In summary, we use aggregated district-level dengue incidence time series data from 2001 to 2019 (inclusive). We illustrate end-to-end operation of the pipeline by training a SARIMAX model on synthetic dengue incidence data (Figure S3).

## Discussion

As climate-driven infectious diseases are increasing in their frequency and intensity, forecasting models play an important role in the design of pre-emptive and reactive interventions. For climate-sensitive diseases, there is currently a gap in the ability to rapidly integrate heterogeneous large-scale datasets into probabilistic models for timely and continuous risk assessment ^10^. To address this gap, we developed a scalable, modular, and open-access pipeline for the ingestion, integration, correction and aggregation of tabular (wealth index) and gridded imagery data. The tool is modular by design and thus extendable to other data types and geographical contexts and improves the ability to perform interdisciplinary analyses of data before, during and ahead of outbreaks of infectious diseases. Beyond that, however, the pipeline is applicable to other research applications that involve the use of weather, climate, and socio-economic data sources such as monitoring changes and drivers in biodiversity.

Best practices in data aggregation and integration for informing control and prevention strategies include several priority areas, such as interdisciplinary collaborations, protocols for sustainable data, reproducible and user-friendly digital tools. Similarly, Ryan *et al.* ^10^ recommended that tools for climate-sensitive infectious diseases should incorporate both climate and epidemiological data, be transparently described and validated, be named, and be accessible and open-access. We sought to incorporate such practices throughout our research, leading to our framework which we believe reflects the 3-U (useful, usable, and used) research framework proposed for adoptable and sustainable digital prediction tools ^33^. In particular, our common data model for integration of data types facilitates downstream analyses of these data together with locally acquired epidemiological data, thus enhancing comparability across settings (as per the FAIR principles ^34^). While we have made an effort to make as much data available via this pipeline, not all processing can be automated and data be redistributed. For example, a user must request API access to the ERA5 climate data to deploy the model locally. Further, restrictions on redistribution of data might exist and change in the future, even when their intended use is for research purposes only. We therefore cannot provide data with redistribution restrictions via this pipeline (see Table S1 for licenses).

The selection of variables in this first version of the pipeline is not exhaustive. However, other variables such as land cover type or mosquito suitability can be easily added to the pipeline and we provide instructions on how to do so in our documentation ( https://dart-pipeline.readthedocs.io/en/latest/reference/custom_metrics.html). Future work may seek to incorporate automated or semi-automated (with user input) variable selection and thus, model selection procedures for dengue prediction models. These selection procedures would likely involve statistically rigorous metrics, e.g. predictive/information criteria and scoring rules ^35– 37^, which could allow different models for different users, regions, and times of the year. Other future work may incorporate explainability metrics ^38– 40^ to automatically capture how individual covariates (both climatic and socio-demographic) influence model predictions, thus providing further transparency for the user in understanding data-driven model processes (data ingestion, model training, and prediction) and important epidemic dynamics and drivers.

While we acknowledge that data integration is only one part of gaining a more comprehensive understanding into the complex transmission dynamics of climate-sensitive infectious diseases, they are critical for robust downstream analyses and reproducibility, especially in settings where automation and re-generation of prediction/forecasting results are required, such as probabilistic forecasting for an outbreak. However, epidemiological data are frequently not openly accessible and biases in them are often unique and depend on the local surveillance systems. Our tool enables standardised integration of data types for drivers of transmission for local partners but today does not accommodate tools for correcting for possible delays in reporting or missing data. However, possible extensions can be easily integrated in future releases ^41, 42^. We have developed our pipeline to be flexible and modular in order to facilitate integration in analysis pipelines. For that we plan to formally integrate the pipeline into applications of dengue modelling in Vietnam and other countries globally.

Multi-modal data integration is a necessary step for robust analyses of disease outbreaks. Our tool is a first step in a platform for data integration and analyses that we believe contributes to future epidemic and pandemic preparedness ^43^, including monitoring and mitigating the impact of climate on infectious diseases.

## Ethics statement

This study did not require ethical approval as it did not involve human participants, human data, or animals. All data used in the pipeline were derived from climate and population datasets. Therefore, no ethical issues are associated with the use of the data.

## Data Availability

Source data for the pipeline is publicly available, see below Source code for the DART-Pipeline is available on GitHub ( https://github.com/kraemer-lab/DART-Pipeline) under MIT license. Source code for dart-bias-correct ( https://github.com/DART-Vietnam/dart-bias-correct) and dart-runner ( https://github.com/DART-Vietnam/dart-runner) are available on GitHub under the GPL-3.0 license. Most source datasets are freely available, except those for forecast bias correction that relies on historical forecast data from ECMWF’s MARS service. ERA5 data at https://cds.climate.copernicus.eu/datasets/reanalysis-era5-single-levels?tab=overview is downloaded using the *cdsapi* Python package (user authentication required). ECMWF weather forecast data is accessed from ECMWF open data website ( https://data.ecmwf.int/forecasts/) using the *ecmwf.opendata* Python package. Worldpop population density data is available at https://hub.worldpop.org/geodata/listing?id=75. Meta relative wealth index is available at https://data.humdata.org/dataset/relative-wealth-index. Geospatial data is obtained from the GADM project at https://gadm.org. Code to reproduce figures is at https://doi.org/10.5281/zenodo.17433818. The data that support the findings of this study are available from authors upon reasonable request by emailing abhishek.dasgupta@dtc.ox.ac.uk or iago.perezfernandez@eng.ox.ac.uk. This article has supplementary information located at https://doi.org/10.5281/zenodo.17565722, containing the following figures and tables: Figure S1. Map of administrative boundaries of the 24 districts (Global Administrative Level 2) of the HCMC province (Global Administrative Level 1), Vietnam, in 2020. Figure S2. Reported dengue incidence in HCMC, Vietnam, from 2000 to 2022. Figure S3. Predicted dengue incidence from SARIMAX models trained using synthetic case incidence data and ERA5 data zonally aggregated from 2001—2019. Table S1. Resampling methods and aggregation schemes for each dataset and variable, used to achieve a common resolution for dengue modelling. Table S2. Formulae for spatial aggregation used by exactextract Table S3. Automated validation checks by variable
