# Supplementary material for: Scalable, open-access and multidisciplinary data integration pipeline for climate-sensitive diseases
Source: Wellcome Open Res. 2026 Aug 1;10:467. Originally published 2025 Aug 29. [Version 3] doi: 10.12688/wellcomeopenres.24774.3 (PMC12648037; doi:10.12688/wellcomeopenres.24774.3)
Supplement: Peer review history

# Reviewer report: Reviewer response for version 2

**DOI:** 10.21956/wellcomeopenres.27767.r140282

**Published:** 2026-01-02

**Authors:** Benn Sartorius

**Affiliations:** 1 The University of Queensland, Brisbane, Australia

Excellent paper and much needed resource.

 Few minor comments and suggestions below:

 Introduction- in addition to scientific/research rationale for data integration, may also want to highlight that it is difficult technically. Also more specifics needed related to heterogeneity of spatial-temporal resolutions, heterogeneity across data types etc. Currently not sufficiently covered in the introduction I think. Same relates to importance of bias correction.

 When referring to existing platforms such as GE, in addition to data types/propriety issues, may need to elaborate more on current limitations in data integration, processing and automation?

Are the conclusions about the tool and its performance adequately supported by the findings presented in the article?

Yes

Is the rationale for developing the new software tool clearly explained?

Yes

Is the description of the software tool technically sound?

Yes

Are sufficient details of the code, methods and analysis (if applicable) provided to allow replication of the software development and its use by others?

Yes

Is sufficient information provided to allow interpretation of the expected output datasets and any results generated using the tool?

Yes

Reviewer Expertise:

Infectious disease epidemiology and modelling, burden of disease

I confirm that I have read this submission and believe that I have an appropriate level of expertise to confirm that it is of an acceptable scientific standard.

# Response

**Published:** 2026-07-08

**Authors:** Abhishek Dasgupta

**Affiliations:** Doctoral Training Centre, University of Oxford, Oxford, England, UK

Thank you for the positive review, apologies for the delayed response. We have updated the text, highlighting the applicability of the pipeline to heterogeneous data, both across data types and spatiotemporal resolutions, and bias-correction functionality which is essential for precipitation analysis.

# Reviewer report: Reviewer response for version 2

**DOI:** 10.21956/wellcomeopenres.27767.r139748

**Published:** 2025-11-25

**Authors:** Oliver Brady

**Affiliations:** 1 London School of Hygiene & Tropical Medicine, London, UK

Approved.

Are the conclusions about the tool and its performance adequately supported by the findings presented in the article?

Partly

Is the rationale for developing the new software tool clearly explained?

Partly

Is the description of the software tool technically sound?

Yes

Are sufficient details of the code, methods and analysis (if applicable) provided to allow replication of the software development and its use by others?

Partly

Is sufficient information provided to allow interpretation of the expected output datasets and any results generated using the tool?

Yes

Reviewer Expertise:

NA

I confirm that I have read this submission and believe that I have an appropriate level of expertise to confirm that it is of an acceptable scientific standard.

# Reviewer report: Reviewer response for version 2

**DOI:** 10.21956/wellcomeopenres.27767.r139747

**Published:** 2025-11-22

**Authors:** Kayode Oshinubi

**Affiliations:** 1 Northern Arizona University, Flagstaff, Arizona, USA

No furtehr comments for the authors.

Are the conclusions about the tool and its performance adequately supported by the findings presented in the article?

Yes

Is the rationale for developing the new software tool clearly explained?

Yes

Is the description of the software tool technically sound?

Yes

Are sufficient details of the code, methods and analysis (if applicable) provided to allow replication of the software development and its use by others?

Yes

Is sufficient information provided to allow interpretation of the expected output datasets and any results generated using the tool?

Yes

Reviewer Expertise:

computational epidemiology, vector-borne diseases, infectious disease modeling

I confirm that I have read this submission and believe that I have an appropriate level of expertise to confirm that it is of an acceptable scientific standard.

# Reviewer report: Reviewer response for version 1

**DOI:** 10.21956/wellcomeopenres.27294.r134098

**Published:** 2025-10-08

**Authors:** Kayode Oshinubi

**Affiliations:** 1 Northern Arizona University, Flagstaff, Arizona, USA

The authors have done a good job by developing this open access pipeline to help improve the predictability and forecast of vector-borne diseases, which are climate sensitive. As someone who is working on vector-borne disease and interested in using climate forecasts in my model, I find reading this article interesting and novel.

 I do not have major revisions for the authors. I want the authors to clarify the following:

 1. Most modelers like to use R software packages. Is there any plans to have an R version of this pipeline?

 2. I strongly believe that there should be more details about the spatial resolution in which the datasets are available. The temporal resolution is very clear. For instance, in the USA, we have CBGs, counties, ZCTAs, etc. Do we have the data in this spatial scale?  Furthermore, in terms of aggregation, how can we aggregate to zip codes or ZCTA resolution?

 Aside from the above-stated minor revisions, I am strongly in support of this article, which will be an added value to the research community, most especially those interested in using these datasets in their research.

Are the conclusions about the tool and its performance adequately supported by the findings presented in the article?

Yes

Is the rationale for developing the new software tool clearly explained?

Yes

Is the description of the software tool technically sound?

Yes

Are sufficient details of the code, methods and analysis (if applicable) provided to allow replication of the software development and its use by others?

Yes

Is sufficient information provided to allow interpretation of the expected output datasets and any results generated using the tool?

Yes

Reviewer Expertise:

computational epidemiology, vector-borne diseases, infectious disease modeling

I confirm that I have read this submission and believe that I have an appropriate level of expertise to confirm that it is of an acceptable scientific standard.

# Response

**Published:** 2025-10-24

**Authors:** Abhishek Dasgupta

**Affiliations:** Doctoral Training Centre, University of Oxford, Oxford, England, UK

The authors have done a good job by developing this open access pipeline to help improve the predictability and forecast of vector-borne diseases, which are climate sensitive. As someone who is working on vector-borne disease and interested in using climate forecasts in my model, I find reading this article interesting and novel.  

**Response**: We thank the reviewer for their positive assessment of our work.  

 I do not have major revisions for the authors. I want the authors to clarify the following:  

 1. Most modelers like to use R software packages. Is there any plans to have an R version of this pipeline?  

**Response:** We chose to use Python for its extensive library support for data science and machine learning and familiarity with the development team. While the library is written in Python, we provide a command line interface as the primary method of interaction with the pipeline that can be used to fetch and process data without knowledge of Python. In the dart-runner repository ( https://github.com/DART-Vietnam/dart-runner), we provide an example integration with a machine learning model written in R. 

 2. I strongly believe that there should be more details about the spatial resolution in which the datasets are available. The temporal resolution is very clear. For instance, in the USA, we have CBGs, counties, ZCTAs, etc. Do we have the data in this spatial scale?  Furthermore, in terms of aggregation, how can we aggregate to zip codes or ZCTA resolution?  

**Response: **The primary spatial resolution in our study is determined by the Worldpop rasters used for population weighted aggregation that are 1km x 1km (see Methods, Spatial aggregation). Raster data is upsampled to this resolution and aggregated over GADM shapefile polygons at admin level 2. Users can provide their own shapefiles, so it would be possible to aggregate to the above mentioned resolutions provided appropriate shapefiles. Our pipeline is modular allowing extensibility to other data sources in the future.  

 Aside from the above-stated minor revisions, I am strongly in support of this article, which will be an added value to the research community, most especially those interested in using these datasets in their research.  

**Response:** We thank the reviewer for their positive feedback and support..

# Reviewer report: Reviewer response for version 1

**DOI:** 10.21956/wellcomeopenres.27294.r132631

**Published:** 2025-09-27

**Authors:** Oliver Brady

**Affiliations:** 1 London School of Hygiene & Tropical Medicine, London, UK

This article describes the development of a pipeline to ingest, bias correct and aggregate climate data- all necessary but fairly limited steps in the longer process of analysing, modelling and forecasting dengue case count data.

 Major comments:

 The authors need to make a clearer case for why existing GIS platforms do not met the needs of users and what advantages this software pipeline brings. Practically this could mean expanding the introduction to clearly articulate the gap that this software tries to fill and providing additional results from the user testing giving quantitative evidence of (e.g. faster run times, improved usability, etc, etc) ideally benchmarked against existing software.

 The unit testing section is currently vaguely described with some important details missing. Specifically who were the users for the unit testing and how were they chosen, what data were collected and how was it analysed (general methods are mentioned, but specific details around what e.g. “regression testing” involves should be detailed). 

 No detail is provided about the relative wealth index from meta nor any necessary processing or validation steps. Typically, local users might want to use local census data to quantify socio-economic conditions. Is this possible within your framework.

 While the software does have the capability to ingest epidemiological data its functionality is quite limited for any operational use of this type of data. No mention is included of how the system would handle data gaps, delays or anomalies- all common features of real-time epidemiological data that are necessary to address prior to developing an operational forecast. Is the intention for this software to only be used for historical analyses or are there plans to develop such functionality- and thus update the software description in this article? 

 The introduction pitches DART as a fairly broad one-stop-shop for “integration of diverse spatio-temporal epidemiological, socio-economic, and climatic datasets”, however this article only demonstrates processing of spatiotemporally complete raster reanalysis data- probably the least complex data type (in terms of completeness, resolution, timeliness, etc) that users might want to work with many of the functionality needed to work with the other datasets missing. I wonder if presenting this as a more modest step in a wider modular framework that works towards this goal may be more appropriate. This might necessitate changing the title and parts of the introduction to focus more on the specific advantages of this pipeline for efficiently processing climate data.

 Minor comments:

 I’m fairly sure this functionality is included, but would be worth mentioning that users can provide their own shapefiles rather than relying on GADM and ISO3 standards. Administrative boundaries frequently change (as pointed out here in this paper) and are disputed so better for local users to have the flexibility to use their own shapefiles

 Suggest making it clear in figure 1 that the dengue prediction model is not (currently?) part of this software

 Could you clarify if the ERA5 and ECMWF weather forecasts are bias corrected separately or as a combined product? – I image the bias correction for long-term climate and short term weather forecasts could give quite different results. 

 While I appreciate the sensitivities that prevent sharing of any dengue data it might be useful to include a dummy dataset or one based on publicly available data to demonstrate end-to-end functionality.

Are the conclusions about the tool and its performance adequately supported by the findings presented in the article?

Partly

Is the rationale for developing the new software tool clearly explained?

Partly

Is the description of the software tool technically sound?

Yes

Are sufficient details of the code, methods and analysis (if applicable) provided to allow replication of the software development and its use by others?

Partly

Is sufficient information provided to allow interpretation of the expected output datasets and any results generated using the tool?

Yes

Reviewer Expertise:

Dengue epidemiology, modelling, spatial analysis.

I confirm that I have read this submission and believe that I have an appropriate level of expertise to state that I do not consider it to be of an acceptable scientific standard, for reasons outlined above.

# Response

**Published:** 2025-10-24

**Authors:** Abhishek Dasgupta

**Affiliations:** Doctoral Training Centre, University of Oxford, Oxford, England, UK

The authors need to make a clearer case for why existing GIS platforms do not met the needs of users and what advantages this software pipeline brings. Practically this could mean expanding the introduction to clearly articulate the gap that this software tries to fill and providing additional results from the user testing giving quantitative evidence of (e.g. faster run times, improved usability, etc, etc) ideally benchmarked against existing software.

**Response**: We thank the reviewer for their feedback. In the introduction we have listed other GIS tools and reference a recent review that has provided an initial list ( https://www.thelancet.com/journals/lanplh/article/PIIS2542-5196(23)00056-6/fulltext) with additional discussion about the importance of data integration here ( https://www.annualreviews.org/content/journals/10.1146/annurev-ento-040124-015101). While some GIS platforms, such as Google Earth Engine exist, they focus on one particular data type, satellite imagery. We build on previous tools and stick together multiple processes including: Ingest: fetch raster data or read tabular (CSV) data, Preprocess: mask, compute derived variables; Aggregate: temporal rollups (weekly/monthly), group-by summaries, Join: Resample, zonal statistics; Output: push to database.  

 The unit testing section is currently vaguely described with some important details missing. Specifically who were the users for the unit testing and how were they chosen, what data were collected and how was it analysed (general methods are mentioned, but specific details around what e.g. “regression testing” involves should be detailed).   

**Response**: We did not perform user testing (other than members of the team replicating the workflow in their own compute environments and performing it across multiple commonly used systems), unit testing here refers to testing units of the code. “Regression testing” refers to the process of ensuring that outputs of the pipeline do not change. These are best practices for developing research software ( https://doi.org/10.1016/j.patter.2021.100206).  

 No detail is provided about the relative wealth index from meta nor any necessary processing or validation steps. Typically, local users might want to use local census data to quantify socio-economic conditions. Is this possible within your framework.

**Response:** In our pipeline, the Relative Wealth Index (RWI) is calculated by aggregating Meta’s wealth estimates, provided as tabular point-level data indexed by quadkeys, to administrative units using population-weighted averages. Population density at ~2.4 km resolution (matching the RWI data resolution) is taken from Meta’s population dataset and used as weights. Any missing or unmatched pixels are excluded through the inner join during merging, so only overlapping data contribute to the aggregation. Within our framework, users can replace Meta’s population layer with local census data aggregated to the required resolution, and this could be further expanded to directly incorporate tabular census data by linking it to administrative boundaries within the pipeline. We updated the manuscript to describe this process clearly and reference the relevant code module.  

 While the software does have the capability to ingest epidemiological data its functionality is quite limited for any operational use of this type of data. No mention is included of how the system would handle data gaps, delays or anomalies- all common features of real-time epidemiological data that are necessary to address prior to developing an operational forecast. Is the intention for this software to only be used for historical analyses or are there plans to develop such functionality- and thus update the software description in this article? 

**Response:** The key contribution of this paper is in providing a standardised pipeline framework for pre-processing various kinds of data (tabular and gridded) that can be used in the analysis of climate-sensitive infectious diseases together with epidemiological data. We recognise that epidemiological data are a key part of any analysis but are often heterogeneous and inaccessible to researchers outside the agency or country where they are collected. Further, epidemiological data are biased in many unpredictable ways that would require deep understanding of the surveillance systems. However, some progress has been made to deal with reporting delays and gaps. Data imputation could be performed using time series foundation models ( https://arxiv.org/abs/2411.07207) and delays in reporting has been discussed here ( https://journals.plos.org/plosntds/article?id=10.1371/journal.pntd.0012501 and https://journals.plos.org/ploscompbiol/article?id=10.1371/journal.pcbi.1012520).   Our framework is able to accommodate these tools and we have now mentioned possible extensions in the discussion: “Epidemiological data is often inaccessible and biases in them are often unique and depend on the local surveillance systems. Our tool enables standardised integration of data types for drivers of transmission for local partners but today does not accommodate tools for correcting for possible delays in reporting or missing data. However, possible extensions can be easily integrated in future releases.”  

 The introduction pitches DART as a fairly broad one-stop-shop for “integration of diverse spatio-temporal epidemiological, socio-economic, and climatic datasets”, however this article only demonstrates processing of spatiotemporally complete raster reanalysis data- probably the least complex data type (in terms of completeness, resolution, timeliness, etc) that users might want to work with many of the functionality needed to work with the other datasets missing. I wonder if presenting this as a more modest step in a wider modular framework that works towards this goal may be more appropriate. This might necessitate changing the title and parts of the introduction to focus more on the specific advantages of this pipeline for efficiently processing climate data.  

**Response: **We thank the reviewer for their comment. Our tool is not only able to integrate raster re-analysis data but also tabular data (see RWI described above). See previous comment about integration of tools for epidemiological data. We have adapted the introduction and discussion to reflect that our tool is a step into the direction of a modular framework and thank the reviewer for this suggestion.  

**Minor comments**:   I’m fairly sure this functionality is included, but would be worth mentioning that users can provide their own shapefiles rather than relying on GADM and ISO3 standards. Administrative boundaries frequently change (as pointed out here in this paper) and are disputed so better for local users to have the flexibility to use their own shapefiles  

**Response:** We have added text to the manuscript and online documentation (https://dart-pipeline.readthedocs.io/en/latest/workflow/using_custom_shapefiles.html) clarifying that users can provide custom configuration to use their own shapefiles.  

 Suggest making it clear in figure 1 that the dengue prediction model is not (currently?) part of this software  

**Response**: We have updated figure 1 to indicate that the model is an external module.  

 Could you clarify if the ERA5 and ECMWF weather forecasts are bias corrected separately or as a combined product? – I image the bias correction for long-term climate and short term weather forecasts could give quite different results. 

**Response**: ERA5 data and ECMWF weather forecasts are corrected using the same technique, but applied differently:   First, ERA5 is corrected following the same approach as in Refs 22,24 of the main article, this is, the straightforward application of quantile mapping using Vietnam gridded precipitation data as reference. Second, real time weather forecasts use ERA5 as reference  and in contrast to the latter, they have an extra dimension compared to ERA5 data as it shows different possible states of the atmosphere in the future (i.e., ensemble members). Each ensemble member was calibrated individually against ERA5. A sentence describing the different application of the bias-correction technique between weather forecast and ERA5 data was added to the main manuscript.  

 While I appreciate the sensitivities that prevent sharing of any dengue data it might be useful to include a dummy dataset or one based on publicly available data to demonstrate end-to-end functionality.

**Response**: We have added a dummy epidemiological dataset that utilises output data from the pipeline and a simple model (SARIMAX) to show end-to-end functionality at https://github.com/DART-Vietnam/DART-manuscript-figures. We have also added a Figure showing predictions from this simple model in the Supplementary Material.

# Response

**Published:** 2025-11-24

**Authors:** Oliver Brady

**Affiliations:** Infectious Disease Epidemiology and Dynamics, London School of Hygiene and Tropical Medicine Faculty of Epidemiology and Population Health, London, England, UK

The authors have adequately addressed my comments
